# Supplementary material for: Benchmarking DNA foundation models for genomic and genetic tasks
Source: Nat Commun. 2025 Nov 28;16:10780. doi: 10.1038/s41467-025-65823-8 (PMC12663285; doi:10.1038/s41467-025-65823-8)

Supplementary Note 1: Sequence classification dataset naming and source

Among the 57 sequence classification datasets, we named the datasets in a manner that clearly shows their features, and we used our naming throughout our study. Specifically:

For **4mc sites detection in multiple species**, we named the six datasets as: A.Thaliana 4mC, C.Elegans 4mC, D.Melanogaster 4mC, E.Coli 4mC, G.Pickeringii 4mC, G.Subterraneus 4mC.

For **DNase-I hypersensitive sites detection**, we named this dataset as DNase_I Hypersensitive.

For **5mC and 6mA modifications detection**, we named the two datasets as: 5-methylcytosin (5mC), N6-methyladenosine (6mA)

For **Promoter identification in multiple species**, we named the eight datasets as: Promoter GM12878, Promoter HUVEC, Promoter Hela-S3, Promoter NHEK, Promoter B_amyloliquefaciens, Promoter R_capsulatus, Promoter Arabidopsis NonTATA, Promoter Arabidopsis TATA.

For **Genomic Benchmarks Dataset Collection**, we downloaded seven datasets from https://github.com/ML-Bioinfo-CEITEC/genomic_benchmarks: demo_coding_vs_intergenomic_seqs, demo_human_or_worm, human_enhancers_cohn, human_enhancers_ensembl, human_ensembl_regulatory, human_nontata_promoters, human_ocr_ensembl. We re-named the seven datasets as: Coding, Human vs Worm, Enhancers Cohn, Enhancers Ensembl, Regulatory Region Type, Promoter NonTATA 251bps, Open chromatin region, respectively.

Besides, we have renamed the rest 33 datasets adopted from DNABERT-2 and NT-v2, in our study for better overall clarity. This document provides a mapping between the original dataset names and the nomenclature in our study. It can serve as a reference for tracing back to the original dataset sources.

Datasets adopted from DNABERT-2

Detailed descriptions of these datasets can be found in the original DNABERT-2 publication.

Our Dataset Naming	Original Reference
Promoter NonTATA 300 bps<br>Promoter TATA 300 bps<br>Promoter All 300 bps	Promoter detection (Human)
Promoter NonTATA 70 bps<br>Promoter TATA 70 bps<br>Promoter All 70 bps	Core promoter detection (Human)
Human TFBS 1<br>Human TFBS 2<br>Human TFBS 3<br>Human TFBS 4<br>Human TFBS 5	Transcription factor binding site prediction (Human)
Mouse TFBS 1<br>Mouse TFBS 2<br>Mouse TFBS 3<br>Mouse TFBS 4<br>Mouse TFBS 5	Transcription factor binding site prediction (Mouse)

Yeast H3<br>Yeast H3K79me3<br>Yeast H3K9ac<br>Yeast H3K14ac<br>Yeast H3K4me3<br>Yeast H3K36me3<br>Yeast H3K4me2<br>Yeast H4<br>Yeast H3K4me1<br>Yeast H4ac	Epigenetic marks prediction (Yeast)
Covid variants	Covid variant prediction (Virus)
Splice Site Type DNABERT	Splice site prediction (Human)

2. Datasets adopted from NT-v2

Detailed descriptions can be found in the corresponding sections of the original NT-v2 publication.

**Our Dataset Naming**	**Original Reference**
Enhancer<br>Enhancer strength	Enhancer sequence prediction, Section A 4.3
Splice Site Type NT<br>Donors<br>Acceptors	Splice site prediction, Section A 4.4

Supplementary Note 2: Sequence classification data notes

Among the total 57 sequence classification datasets, a few of them may **not** be fully relied on experimental evidence or gold standard sources (for example, ENCODE, MethSMRT, etc.), but contains some artificial sequences. Below is the table listing the properties of each dataset. Please check the original sources of datasets for more details.

**Dataset Name**	**is** relied on experimental evidence or gold standard sources
5mC	Yes
6mA	Yes
A.thaliana 4mC	Yes
C.elegans 4mC	Yes
D.melanogaster 4mC	Yes
E.coli_4mC	Yes
G.pickeringii 4mC	Yes
G.subterraneus 4mC	Yes
DNase_I Hypersensitive	Yes
Human TFBS 1	Yes
Human TFBS 2	Yes
Human TFBS 3	Yes
Human TFBS 4	Yes
Human TFBS 5	Yes
Promoter GM12878	Yes
Promoter HUVEC	Yes
Promoter Hela-S3	Yes
Promoter NHEK	Yes
Promoter Arabidopsis NonTATA	Yes
Promoter Arabidopsis TATA	Yes
Promoter B_amyloliquefaciens	Yes
Promoter R_capsulatus	Yes
Acceptors	Yes
Coding	Yes
Covid Variants	Yes
Donors	Yes
Enhancer	No
Enhancer Strength	No
Enhancer Cohn	Yes
Enhancer Ensembl	Yes
Human vs Worm	Yes
Mouse TFBS 1	No
Mouse TFBS 2	No
Mouse TFBS 3	No
Mouse TFBS 4	No
Mouse TFBS 5	No
Open Chromatin Region	Yes

Promoter All 300bps	Yes
Promoter All 70bps	Yes
Promoter NonTATA 251bps	Yes
Promoter NonTATA 300bps	Yes
Promoter NonTATA 70bps	Yes
Promoter TATA 300bps	Yes
Promoter TATA 70bps	Yes
Regulatory Region Type	Yes
Splice Site Type DNABERT	Yes
Splice Site Type NT	Yes
All Yeast Datasets	Unknown

Supplementary Table 1: Optimal pooling methods in terms of AUC out of 52 total binary sequence classification datasets for all DNA Foundation Models, with one-sided Delong's Test p<0.01.

Model	Mean Pooling	Max Pooling	Summary Token	None
DNABERT-2	43 (75.0%)	0 (0.0%)	1 (1.9%)	8 (15.4%)
NT-v2	42 (80.8%)	0 (0.0%)	2 (3.8%)	8 (15.4%)
HyenaDNA	35 (67.3%)	3 (5.8%)	0 (0.0%)	14 (26.9%)
Caduceus-Ph	37 (71.2%)	2 (3.8%)	2 (3.8%)	11 (21.2%)
GROVER	41 (78.8%)	0 (0.0%)	0 (0.0%)	11 (21.2%)

Supplementary Table 2: The accuracy for all multi-class classification datasets in this study. ***Bolded: row maximum with 0.01 tolerance.***

Data	DNABERT-2	NT-v2	HyenaDNA	Caduceus-Ph	GROVER
Enhancer Strength	**0.715**	0.653	0.69	**0.713**	0.703
Splice Site Type, NT	0.496	0.518	**0.563**	0.516	0.515
Splice Site Type, DNABERT-2	0.605	0.601	0.618	**0.628**	0.611
Covid Variants	**0.664**	0.494	0.629	0.609	**0.659**
Regulatory Region Type	0.676	0.644	**0.83**	0.66	0.615

Supplementary Table 3: Random Forest and XGBoost regression performance for gene expression prediction, averaged across all genes. P-value is comparison between random forest and XGBoost results, using two-sided Wilcoxon signed-rank test.

**Model**	**Input Length**	**Metric**	**Random Forest Mean**	**XGBoost Mean**	**p-value**
DNABERT-2	6000 bp	Correlation	**0.1208**	0.0959	$< 10^{-16}$
		MSE	**0.2362**	0.2676	$< 10^{-16}$
NT-v2	6000 bp	Correlation	**0.1223**	0.1038	$< 10^{-16}$
		MSE	**0.2355**	0.2579	$< 10^{-16}$
HyenaDNA	6000 bp	Correlation	**0.1224**	0.1027	$< 10^{-16}$
		MSE	**0.2349**	0.2579	$< 10^{-16}$
Caduceus-Ph	6000 bp	Correlation	**0.1229**	0.1024	$< 10^{-16}$
		MSE	**0.2340**	0.2580	$< 10^{-16}$
GROVER	2048 bp	Correlation	**0.1136**	0.1035	$< 10^{-16}$
		MSE	**0.2334**	0.2415	$< 10^{-16}$
Caduceus-Ph Long	~131K bp	Correlation	**0.1267**	0.0947	$< 10^{-16}$
		MSE	**0.2266**	0.2760	$< 10^{-16}$
HyenaDNA Long	~196K bp	Correlation	**0.1369**	0.1014	$< 10^{-16}$
		MSE	**0.2257**	0.2700	$< 10^{-16}$
Enformer	~196K bp	Correlation	**0.1294**	0.0901	$< 10^{-16}$
		MSE	**0.2269**	0.2793	$< 10^{-16}$

Supplementary Table 4: Comparison of short versus long sequence models on the same set of genes. P-value is calculated using two-sided Wilcoxon signed-rank test.

	Correlation (short sequence)	Correlation (long sequence)	p-value
HyenaDNA	0.122	0.137	0.0005
Caduceus-Ph	0.124	0.127	0.975

Supplementary Table 5: Hyperparameter grid for gene expression prediction benchmark

Random Forest

N estimators: 100, 200, 500

max depth: None, 5, 10

XGBoost

N estimators: 100, 200, 500

Max depth: 3, 5, 7

Supplementary Table 6: Hyperparameter grid for sequence classification benchmark

Random Forest

N estimators: 200, 500, 1000

max depth: None, 20

min samples split: 2,5

max features: square root, log 2

Naïve Bayes

Var smoothing: 1e-10. 1e-9. 1e-8

Elastic Net Logistic Regression

C: 0.1, 1, 10

L1 ratio: 0.1, 0.5, 0.9

class weight: balanced, None

Supplementary Table 7: Hyperparameter grids for the random forest in variant effect quantification benchmark

Models with high output dimension (Enformer using either hidden states and outputs, Sei using either hidden states and outputs, AlphaGenome):

N estimators: 100, 200, 400

Max depth: 10, None

Max features: log2, 32,

Min samples leaf: 5, 10

Models with low output dimension (DNABERT-2, NT-v2, GROVER, Caducues-Ph, HyenaDNA, HyenaDNA-450K)

N estimators: 100, 200, 400

Max depth: 10, None

Max features: log2, 32, sqrt

Min samples leaf: 5, 10

Supplementary Table 8: Details of each model. The bolded ones are the included configurations in this study.

Model Configuration	Output Embedding Dimension
**DNABERT-2**	768
NT-v2-50m	512
NT-v2-100m	512
NT-v2-250m	768
**NT-v2-500m**	1024
Hyena-tiny-1k	128
**Hyena-tiny-1k-d256**	256
Hyena-tiny-16k-d128	128
Hyena-small-32k	256
**Hyena-medium-160k**	256
**Hyena-medium-450k**	256
Hyena-large-1m	256
**GROVER**	768
**Caduceus-Ph-131k-d-256**	256
Caduceus-Ph-1k-d-256	256
Caduceus-Ph-1k-d-128	118

Supplementary Figure 1: Classifier comparison, for all models and pooling methods. We calculate AUC scores from all 52 binary sequence classification datasets included in this study. Boxplots show the median (center line), interquartile range (box = 25th–75th percentiles), and whiskers correspond to 1.5 × interquartile range. minima and maxima correspond to the whisker ends.

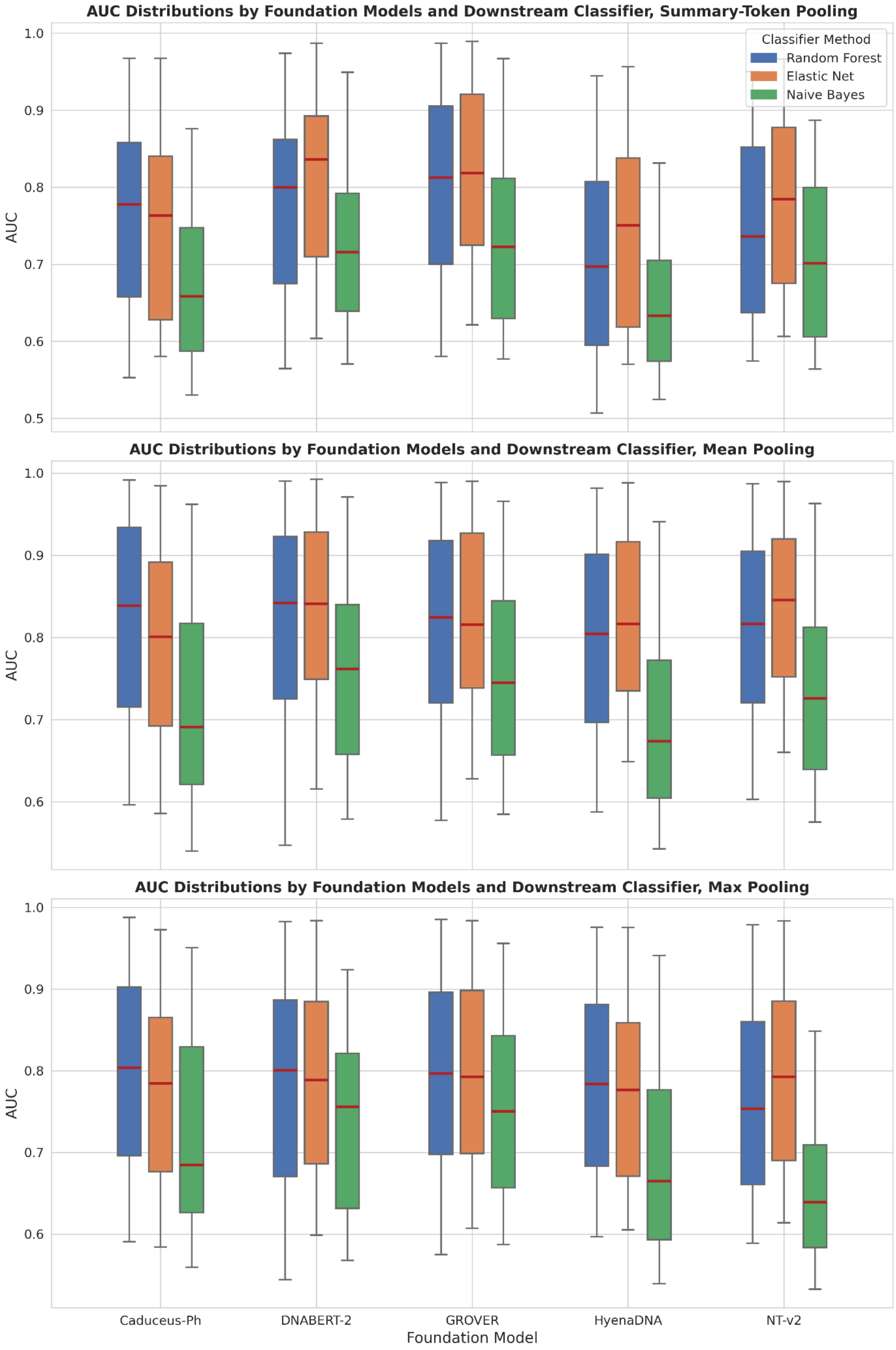

Supplementary Figure 2: The heatmap of the difference between attention matrices for TAD-centered versus background sequences. The horizontal axis represents key tokens, and the vertical axis represents query tokens. Each point (x,y) shows the difference in attention weight that query token y places on key token x when comparing TAD-centered versus background sequences. The central 400 tokens (approximately positions 200-600) correspond to the TAD region. If NT-v2 recognized TAD boundaries, we would expect a vertical band of positive differences in this central region.

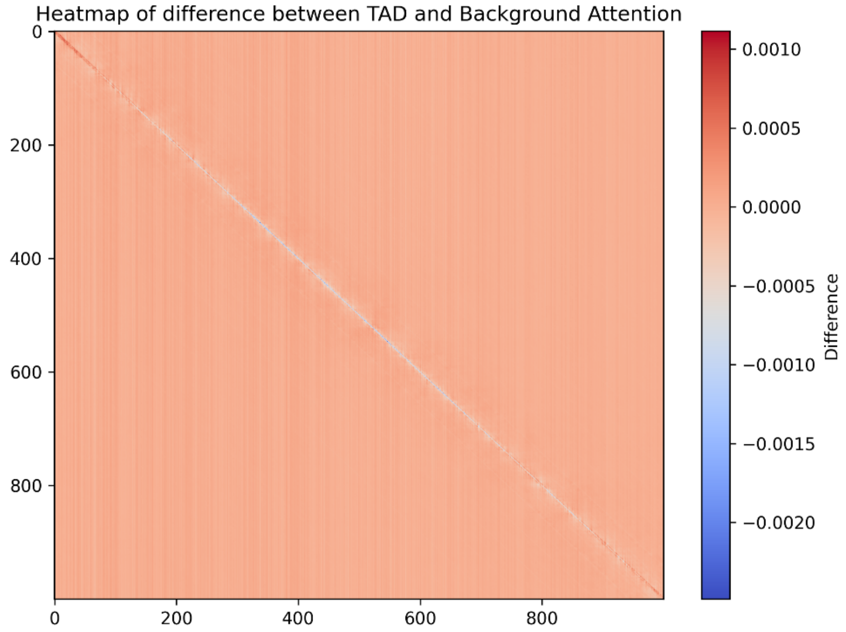

Supplement: Supplementary file 1 — Supplementary Information [file 41467_2025_65823_MOESM1_ESM.pdf]
