# Supplementary material for: Benchmarking DNA foundation models for genomic and genetic tasks
Source: Nat Commun. 2025 Nov 28;16:10780. doi: 10.1038/s41467-025-65823-8 (PMC12663285; doi:10.1038/s41467-025-65823-8)
Supplement: Supplementary file 2 — Description of Additional Supplementary Files [file 41467_2025_65823_MOESM2_ESM.pdf]

Description of Additional Supplementary Files

Supplementary Data 1: The datasets where the baseline CNN achieves statistically significant higher AUCs than DNA Foundation Models. Test using one-sided Delong's Test $p<0.01$.

Supplementary Data 2: The datasets where the DNA Foundation Models achieves statistically significant higher AUCs than the baseline CNN. Test using one-sided Delong's Test $p<0.01$.

Supplementary Data 3: Top genes ranked by random forest correlation for each model. Gene ids are in their original Ensembl ids as in GTEx dataset.

Supplementary Data 4: The test AUC scores for each model, each test set (independent group of chromosomes) in the variant quantification effect benchmark. From up to bottom: patho, eQTL, sQTL, paQTL, ipaQTL. There are notable differences between different test sets for paQTL and ipaQTL.

Supplementary Data 5: HyenaDNA pretrained on DNABERT-2 multispecies dataset, compared with the HyenaDNA-1K checkpoint. Bolded: one-sided DeLong's test p-value < 0.01.

Supplementary Data 6: Details of each sequence classification benchmark dataset, including sequence length statistics and sample size statistics.
