# Supplementary material for: Benchmarking DNA foundation models for genomic and genetic tasks
Source: Nat Commun. 2025 Nov 28;16:10780. doi: 10.1038/s41467-025-65823-8 (PMC12663285; doi:10.1038/s41467-025-65823-8)
Supplement: Supplementary file 4 — Reporting Summary [file 41467_2025_65823_MOESM4_ESM.pdf]

nature portfolio

Corresponding author(s): Peng Wei, Chong Wu

Last updated by author(s): Oct 14, 2025

Reporting Summary

Nature Portfolio wishes to improve the reproducibility of the work that we publish. This form provides structure for consistency and transparency in reporting. For further information on Nature Portfolio policies, see our Editorial Policies and the Editorial Policy Checklist.

Statistics

For all statistical analyses, confirm that the following items are present in the figure legend, table legend, main text, or Methods section.

n/a	Confirmed	
☐	☒	The exact sample size (*n*) for each experimental group/condition, given as a discrete number and unit of measurement
☐	☒	A statement on whether measurements were taken from distinct samples or whether the same sample was measured repeatedly
☐	☒	The statistical test(s) used AND whether they are one- or two-sided<br>*Only common tests should be described solely by name; describe more complex techniques in the Methods section.*
☐	☒	A description of all covariates tested
☐	☒	A description of any assumptions or corrections, such as tests of normality and adjustment for multiple comparisons
☐	☒	A full description of the statistical parameters including central tendency (e.g. means) or other basic estimates (e.g. regression coefficient) AND variation (e.g. standard deviation) or associated estimates of uncertainty (e.g. confidence intervals)
☐	☒	For null hypothesis testing, the test statistic (e.g. *F*, *t*, *r*) with confidence intervals, effect sizes, degrees of freedom and *P* value noted<br>*Give P values as exact values whenever suitable.*
☒	☐	For Bayesian analysis, information on the choice of priors and Markov chain Monte Carlo settings
☒	☐	For hierarchical and complex designs, identification of the appropriate level for tests and full reporting of outcomes
☐	☒	Estimates of effect sizes (e.g. Cohen's *d*, Pearson's *r*), indicating how they were calculated

Our web collection on statistics for biologists contains articles on many of the points above.

Software and code

Policy information about availability of computer code

Data collection	The raw data relevant to this study are publicly available datasets, and can be found in the following web links:<br>https://bioinfo.uth.edu/Deep4mC/Download.php<br>http://bliulab.net/iDHS-EL/data<br>https://github.com/FakeEnd/iDNA_ABF<br>https://github.com/HaoWuLab-Bioinformatics/iPro-WAEL<br>https://github.com/MAGICS-LAB/DNABERT_2<br>https://huggingface.co/datasets/InstaDeepAI/nucleotide_transformer_downstream_tasks/tree/main<br>https://github.com/ML-Bioinfo-CEITEC/genomic_benchmarks<br>https://huggingface.co/datasets/InstaDeepAI/genomics-long-range-benchmark/tree/main/variant_effect_pathogenic<br>https://console.cloud.google.com/storage/browser/basenji_hic/insulation<br>https://gtexportal.org/home/protectedDataAccess<br>https://www.gtexportal.org/home/downloads/adult-gtex/qtl<br>https://console.cloud.google.com/storage/browser/borzoi-paper/qtl
Data analysis	All the codes used for data analysis in this study have been deposited, and are available on https://github.com/ChongWuLab/dna_foundation_benchmark.

For manuscripts utilizing custom algorithms or software that are central to the research but not yet described in published literature, software must be made available to editors and reviewers. We strongly encourage code deposition in a community repository (e.g. GitHub). See the Nature Portfolio guidelines for submitting code & software for further information.

Data

Policy information about availability of data

All manuscripts must include a data availability statement. This statement should provide the following information, where applicable:

- Accession codes, unique identifiers, or web links for publicly available datasets
- A description of any restrictions on data availability
- For clinical datasets or third party data, please ensure that the statement adheres to our policy

The processed benchmarking datasets generated in this study (excluding the Gene Expression Prediction benchmark) have been deposited in the Hugging Face Hub under https://huggingface.co/datasets/hfeng3/dna_foundation_benchmark_dataset. The Gene Expression Prediction benchmark involves human participant data and is available under restricted access to protect participant privacy; raw individual whole genome sequencing data can be obtained by application via the GTEx Protected Data Access portal (https://gtexportal.org/home/protectedDataAccess); the public aggregated gene-expression releases are available at https://www.gtexportal.org/home/downloads/adult-gtex/qtl. The original datasets used for the sequence-classification benchmarks are available from the sources cited in the main text. The Variant Effect Quantification (pathogenic versus common variant) benchmark dataset is available from the Genomics Long-Range Benchmark repository on the Hugging Face Hub (https://huggingface.co/datasets/InstaDeepAI/genomics-long-range-benchmark/tree/main/variant_effect_pathogenic). The QTL benchmark files were accessed from the Borzoi paper repository (Google Cloud Storage: https://console.cloud.google.com/storage/browser/borzoi-paper/qtl). The TAD region recognition benchmark files were accessed from the Basenji Hi-C repository (Google Cloud Storage: https://console.cloud.google.com/storage/browser/basenji_hic/insulation).

Research involving human participants, their data, or biological material

Policy information about studies with human participants or human data. See also policy information about sex, gender (identity/presentation), and sexual orientation and race, ethnicity and racism.

Reporting on sex and gender	We used sex of samples in GTEx as one of covariates to regress out the individual-level gene expression.
Reporting on race, ethnicity, or other socially relevant groupings	Our research do not involve human participants categorized by race, ethnicity, or other socially relevant groupings.
Population characteristics	We used probabilistic estimation of expression residuals (PEER) factors and genotype principal components in GTEx as covariates to regress out the individual-level gene expression.
Recruitment	Our research do not involve human recruitment.
Ethics oversight	Our research do not involve ethics oversight.

Note that full information on the approval of the study protocol must also be provided in the manuscript.

Field-specific reporting

Please select the one below that is the best fit for your research. If you are not sure, read the appropriate sections before making your selection.

☒ Life sciences ☐ Behavioural & social sciences ☐ Ecological, evolutionary & environmental sciences

For a reference copy of the document with all sections, see nature.com/documents/nr-reporting-summary-flat.pdf

Life sciences study design

All studies must disclose on these points even when the disclosure is negative.

Sample size	We used all available samples for whole blood tissue in GTEx for analysis of gene expression prediction.
Data exclusions	No data was excluded in the study.
Replication	The study spans multiple tasks across multiple DNA foundation models. We fixed random number generating seeds (e.g., Python random, NumPy) in all experiments, and used a deterministic software environment. All experiments were re-run two times with the same seed settings and produced identical results.
Randomization	Samples were randomly divided into train/test splits in GTEx whole blood tissue for analysis of gene expression prediction.
Blinding	Blinding is not relevant, because there is no experimental group study in our study.

Reporting for specific materials, systems and methods

We require information from authors about some types of materials, experimental systems and methods used in many studies. Here, indicate whether each material, system or method listed is relevant to your study. If you are not sure if a list item applies to your research, read the appropriate section before selecting a response.

Materials & experimental systems

n/a	Involved in the study	
☒	☐	Antibodies
☒	☐	Eukaryotic cell lines
☒	☐	Palaeontology and archaeology
☒	☐	Animals and other organisms
☒	☐	Clinical data
☒	☐	Dual use research of concern
☒	☐	Plants

Methods

n/a	Involved in the study	
☒	☐	ChIP-seq
☒	☐	Flow cytometry
☒	☐	MRI-based neuroimaging

Plants

Seed stocks	No plant study was involved.
Novel plant genotypes	No plant study was involved.
Authentication	No plant study was involved.
